# Identification of interactions using model-based multifactor dimensionality reduction

**DOI:** 10.1186/s12919-016-0019-8

**Published:** 2016-10-18

**Authors:** Damian Gola, Inke R. König

**Affiliations:** Institut für Medizinische Biometrie und Statistik, Universität zu Lübeck, Universitätsklinikum Schleswig-Holstein — Campus Lübeck, Ratzeburger Allee 160, Lübeck, 23562 Germany

## Abstract

**Background:**

Common complex traits may involve multiple genetic and environmental factors and their interactions. Many methods have been proposed to identify these interaction effects, among them several machine learning and data mining methods. These are attractive for identifying interactions because they do not rely on specific genetic model assumptions. To handle the computational burden arising from an exhaustive search, including all possible combinations of factors, filter methods try to select promising factors in advance.

**Methods:**

Model-based multifactor dimensionality reduction (MB-MDR), a semiparametric machine learning method allowing adjustment for confounding variables and lower level effects, is applied to Genetic Analysis Workshop 19 (GAW19) data to identify interaction effects on different traits. Several filtering methods based on the nearest neighbor algorithm are assessed in terms of compatibility with MB-MDR.

**Results:**

Single nucleotide polymorphism (SNP) rs859400 shows a significant interaction effect (corrected *p* value <0.05) with age on systolic blood pressure (SBP). We identified 23 SNP–SNP interaction effects on hypertension status (HS), 42 interaction effects on SBP, and 26 interaction effects on diastolic blood pressure (DBP). Several of these SNPs are in strong linkage disequilibrium (LD). Three of the interaction effects on HS are identified in filtered subsets.

**Conclusions:**

The considered filtering methods seem not to be appropriate to use with MB-MDR. LD pruning is further quality control to be incorporated, which can reduce the combinatorial burden by removing redundant SNPs.

## Background

Common complex traits, such as blood pressure, are at least partly based on complex molecular mechanisms likely to involve multiple genetic factors and their interactions. In addition, the extent to which genetic factors are expressed may also depend on interactions with environmental factors, such as age or sex. Factors influencing the trait of interest by nonlinear interaction with other factors may have a small effect on their own, and most traditional methods suffer from the curse of dimensionality as a consequence of small sample sizes.

As a result, several methods have been specifically proposed to identify gene–gene (G × G) and gene-environment (G × E) interactions. Two main classes of methods can be identified: parametric methods, like regression-based approaches, and nonparametric methods, which covers most machine learning and data-mining approaches. One widely used nonparametric method is the multifactor dimensionality reduction (MDR) [1] that has been extended and altered subsequently. Model-based MDR (MB-MDR) [2] is one alteration that bridges the aforementioned classes by combining advantages of nonparametric approaches, that is, no distributional assumptions are imposed, with those of parametric approaches, that is, adjustment for confounding variables and lower level main effects.

To handle the combinatorial challenge of millions to billions of possible factor combinations, filter algorithms have been proposed that select interesting factors in a preprocessing manner.

These approaches are combined in this study in application on Genetic Analysis Workshop 19 (GAW19) data to detect interactions and assess the usability of filter methods.

## Methods

### Data

Data provided by the GAW19 are used [3]. As real phenotype data, we use age, sex, systolic blood pressure (SBP), diastolic blood pressure (DBP), and antihypertensive medication (BPMEDS). We use high-quality hard genotype calls and restrict our analyses to biallelic variants, resulting in 163,622 single-nucleotide polymorphisms (SNPs). Hypertension status (HS) is defined by SBP >140 mmHg and DBP >90 mmHg or BPMEDS usage, yielding 219 cases and 1632 controls. Because of missing phenotype data, 92 samples were excluded.

### Quality control

For quality control, we use a stepwise routine. In every step, samples with a call rate of less than 97 % or deviation from mean heterozygosity of more than three SD are excluded. Likewise, SNPs with a call rate of less than 98 % are excluded, separately for cases and controls, minor allele frequency (MAF) less than 1 %, or a *p* value of less than 0.00001 in a test for deviation from Hardy-Weinberg equilibrium (HWE) in controls. Steps are repeated until no more samples or SNPs are excluded. On the remaining data set principal components are analyzed to find genetic outliers. To find genetic relatives, SNPs are analyzed with R package [4] SNPRelate [5], and identity-by-descent (IBD) and identity-by-state (IBS) estimates are used to determine samples to remove.

### Filtering of single-nucleotide polymorphisms

Filter methods can be applied to reduce the set of possible SNP combinations, thus reducing the computational burden in interaction analyses. These methods aim to select potentially important SNPs for subsequent analyses. We used nearest-neighbor-based filtering methods, namely Relief [6], ReliefF [7], Tuned ReliefF (TuRF) [8], Spatially Uniform ReliefF (SURF) [9], SURF* [10], multiSURF (multiple runs of SURF), and SURF*nTuRF (combination of SURF* and TuRF). Basically, all these filtering methods assign a weight to each SNP based on whether the nearest neighbor(s) of the same affection status and the nearest neighbor(s) of the other affection status group have the same or different genotypes. For further information on these methods, we refer you to the literature. All filtering methods are implemented in the MDR software package (ver. 3.0.2, http://sourceforge.net/projects/mdr/).

We apply all filtering methods to the quality-controlled data to select the 1000 top-weighted SNPs, using HS as affection status. All other parameters are set to default. For continuous traits such as DBP and SBP, these filtering methods are not applicable.

### Model-based multifactor dimensionality reduction

MB-MDR [2] aggregates SNP combinations into risk groups with strong evidence regarding high or low risk of disease. Thus, the high-dimensional space of SNP combinations is reduced to a new 1-dimensional factor to increase the power to detect interactions. Applied to the analysis of SNP-SNP interactions in the Genetic Analysis Workshop (GAW) data the steps of MB-MDR are as follows:

Step 1: Dimensionality reduction.
  1. Select two SNPs with three possible discrete genotypes each.
  2. Represent combination of selected SNPs in 2-dimensional space as cells *c*_*j*_, eg, 0–0, 0–1,…
  3. For *j = 1,…,9,* evaluate significance of association test *T*_*j*_ on *c*_*j*_ versus all other cells. The appropriate test depends on the trait. For HS a *χ* ^2^-test is used; for DBP and SBP a *t*-test is used.
    If *T*_*j*_ is not significant, label *c*_*j*_ as *O* (“no evidence”);
    Else if *T*_*j*_ *> 0,* label *c*_*j*_ as *H*(igh) risk;
    Else label *c*_*j*_ as *L*(ow) risk.
Step 2: Association test on lower-dimensional construct.
  1. Perform association test *T*_*H*_, comparing *H* versus *{L, O}*. Again, selection of association test depends on the trait (see Step 1).
  2. Perform association test *T*_*L*_, comparing *L* versus *{H, O}*. Again, selection of association test depends on the trait (see Step 1).
  3. Select max(*T*_*H*_, *T*_*L*_).
Step 3: Significance assessment.
  1. Repeat Steps 1 and 2 for each possible SNP combination.
  2. Assess significance of each max(*T*_*H*_, *T*_*L*_) by permutation-based maxT multiple-testing correction algorithm [11].

The extension of the algorithm to higher-order interactions is straightforward. Furthermore, one SNP can be replaced by any environmental factor (age, sex) with discrete states to analyze G × E interactions. It should be noted that the term “model-based” in MB-MDR alludes to the use of statistical tests in Steps 1 and 2. For our analyses, we use the C++ implementation of MB-MDR (ver. 4.1.0, http://www.statgen.ulg.ac.be/software.html); for further information on this method, we refer the reader to the literature.

### Genome-wide analysis of main effects

To assess the main effects of SNPs on HS, SBP, and DBP, MB-MDR is used with default settings but switched off interaction. We use a maximum of 100,000,000 permutations and the MAXT option for multiple-testing correction to obtain *p* values. Thus, *p* values are corrected for multiple testing within each of the analyses on HS, SBP, and DBP only. For computational feasibility, after each 100,000 permutations, the upper bound of a 95 % confidence interval, around 1.5 times the inverse of all permutations so far, is calculated. SNPs with an estimated *p* value exceeding this bound are removed from further permutation runs.

### Model-based multifactor dimensionality reduction analyses for gene-environment interactions

We next analyze the interaction effects of all SNPs, using sex and age as environmental factors on HS, SBP, and DBP. Age is categorized into three balanced groups (young, middle, old). For adjustment for main effects, we use the co-dominant method of MB-MDR as suggested by Mahachie John et al [12]. We use 10,000 permutations and the speedMAXT option for multiple-testing correction to obtain *p* values. Again, *p* values are corrected for multiple testing within each of the analyses on HS, SBP, and DBP only. We compare these interaction results with the results from the main effect analyses to ensure that main effects do not drive interaction results.

### Model-based multifactor dimensionality reduction analyses for SNP–SNP interactions

SNP–SNP interaction analyses on HS, SBP, and DBP are performed on the full set of SNPs, as well as on the top 1000 filtered SNPs on HS. If the filters work adequately for MB-MDR, we expect an overlap of identified interactions. We use a maximum of 10,000 permutations and speedMAXT option for multiple-testing correction to obtain *p* values. As before, *p* values are corrected for multiple testing within each of the analyses on HS, SBP, and DBP only.

## Results

In nine runs of quality control, 33 samples were excluded because of deviation from mean heterozygosity. In addition, one sample was excluded after IBD analyses. A total of 1,586,958 SNPs were excluded because of low MAF and 1806 SNPs were excluded because of deviation from HWE in controls. After quality control, 1820 samples (216 cases, 1604 controls) and 46,746 SNPs remained.

**Fig. 1:**
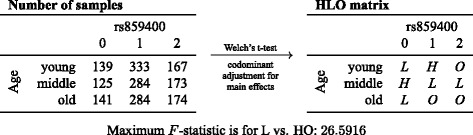
Interaction between age and rs859400 on systolic blood pressure. *Left:* Number of samples per factor combination. *Right:* Labels of cells as (*H*)igh or (*L*)ow risk or unknown (*O*)

Table 1 shows the results of screening for main effects. We identified one SNP with a significant interaction effect with age on SBP. As an example for the internal representation of factor combinations in MB-MDR we refer to Fig. 1, which shows the interaction rs859400 × age on SBP. We see a nonlinear interaction effect, as the risk for a higher SBP at every age depends on a different genotype at rs859400.

**Table 1:** Main effects and G × E interaction effects ^a^

Trait	Env. Factor	SNP	Chr.	Position	Gene	*p* Value
HS	–	rs145441374	13	53 420 325	*PCDH8*	8.135 × 10^−5^
	–	rs1639	19	45 772 798	*DMPK*	1.883 × 10^−5^
	–	rs1991818	19	51 485 013	*KLK7*	4.645 × 10^−5^
DBP	–	rs2073371	21	32 493 204	*TIAM1*	7.396 × 10^−5^
SBP	Age	rs859400	1	175 375 334	*TNR*	0.0415

^a^ Results of model-based multifactor dimensionality reduction (*MB-MDR*) analyses for main effects of single-nucleotide polymorphisms (*SNPs*) and with sex and age (young, middle, old) as environmental factors (*Env. Factor*). Traits are hypertension status (*HS*), systolic blood pressure (*SBP*) and diastolic blood pressure (*DBP*). Interactions with *p* value <0.05 are shown with chromosome (*Chr.*), position and associated gene

**Fig. 2:**
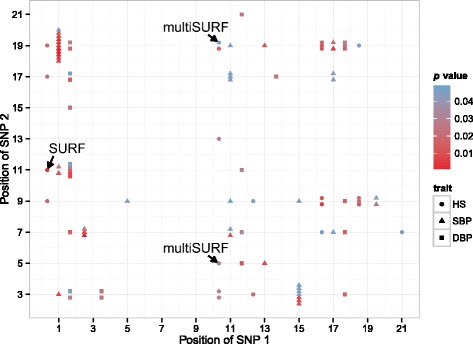
Pairwise SNP–SNP interactions. SNP–SNP interactions with *p* value <0.05 on unfiltered set, grouped by trait (HS, hypertension status; SBP, systolic blood pressure; DBP, diastolic blood pressure). Multiple SNP–SNP interactions on same chromosomes are stacked, *p* values are color-coded and corrected for multiple testing within the analyses on HS, SBP, and DBP. Annotations denote interaction effects that were identified in unfiltered and filtered subsets

Figure 2 shows the results of SNP–SNP interaction analyses on the full set of SNPs. We identified 23 SNP–SNP interactions on HS, 42 interactions on SBP, and 26 interactions on DBP. There are no intersecting interaction effects between traits. Two clusters can be identified for SBP, one involving SNPs on chromosomes 1 and 19 (11 SNP–SNP interaction effects; 6 and 2 SNPs on chromosomes 1 and 19, respectively), another smaller cluster with SNPs on chromosomes 3 and 15 (7 SNP–SNP interaction effects; 3 and 6 SNPs on chromosomes 3 and 15, respectively). SNPs rs4802566 and rs4802565 on chromosome 19 in the first cluster are in strong linkage disequilibrium (LD) (*r* ^*2*^ = 0.9164), as well as several other SNPs on the other chromosomes in both clusters.

Three interaction effects identified on the full set of SNPs are also contained in the filtered sets of SNPs: 1 by SURF, 2 by multiSURF. None of the other filtered sets of SNPs contain SNP pairs showing a *p* value of <0.05 in the full set of SNPs.

## Discussion

We successfully identified nonlinear G × G and G × E interaction effects with MB-MDR. None of the identified main effects triggered an interaction effect. Clearly, these interaction effects need replication to rule out false-positive findings. For this, it might be of interest to compare our results to those of other GAW19 participants. The filtering methods considered did not select SNPs that are involved in the top interaction effects from the unfiltered set. A possible explanation is that the applied filter methods tend to select SNPs with a strong main effect. In contrast, MB-MDR is designed to identify nonlinear interactions, which may or may not have main effects as well.

## Conclusions

Filters based on a nearest-neighbor approach are not suitable for analyses with the MB-MDR method. Based on the full set of SNP data, several SNPs in strong LD are identified involved in interaction effects. Although this is to be expected, this shows that LD pruning is a further quality control to be incorporated as it can reduce the combinatorial burden by removing surrogate SNPs.
